# Drug Reaction With Eosinophilia and Systemic Symptoms (DRESS) Syndrome Following Carbamazepine Use in a Young Male With Psychiatric Comorbidities

**DOI:** 10.7759/cureus.104499

**Published:** 2026-03-01

**Authors:** Nilar Win, Bianca Afroz Liya, Sadia Arefin

**Affiliations:** 1 Acute Medicine, Luton and Dunstable University Hospital, Luton, GBR; 2 General Internal Medicine, Luton and Dunstable University Hospital, Luton, GBR

**Keywords:** carbamazepine, dermatopathic lymphadenitis, dress syndrome, drug hypersensitivity, eosinophilia, rash

## Abstract

Drug reaction with eosinophilia and systemic symptoms (DRESS) is a rare but potentially life-threatening drug-induced hypersensitivity reaction. It is characterized by fever, widespread rash, hematologic abnormalities including eosinophilia, lymphadenopathy, and visceral organ involvement. We report a case of a 25-year-old male who developed DRESS syndrome following initiation of carbamazepine for psychiatric indications. The patient presented with systemic symptoms and a characteristic dermatologic and hematologic profile. Diagnostic workup included lymph node biopsy and imaging, with findings consistent with reactive lymphadenopathy. Prompt recognition and cessation of the offending drug, alongside supportive treatment, led to clinical improvement.

## Introduction

Drug reaction with eosinophilia and systemic symptoms (DRESS) syndrome is a severe adverse drug reaction with delayed onset, typically occurring two to eight weeks after exposure to the offending medication [1]. It is most frequently associated with aromatic anticonvulsants such as carbamazepine and phenytoin, as well as allopurinol and sulfonamides [2]. The exact pathogenesis remains unclear, but proposed mechanisms include drug-specific immune responses, genetic susceptibility, and viral reactivation [3]. Mortality can reach up to 10%, most commonly due to hepatic failure [4]. Early diagnosis and immediate withdrawal of the causative drug are essential to improve outcomes [5].

## Case presentation

A 25-year-old male presented with a one-day history of fever, shortness of breath, pleuritic chest pain, productive cough, and a generalized erythematous, intensely pruritic rash involving the trunk and extremities. He also developed excoriated blistering lesions around the lips, without mucosal ulceration. Past medical history included schizophrenia (recent inpatient admission), asthma, irritable bowel syndrome, attention-deficit/hyperactivity disorder (ADHD), and a history of polysubstance misuse (cannabis, 3,4-methylenedioxymethamphetamine (MDMA), heroin, lysergic acid diethylamide (LSD), and cocaine). Carbamazepine had been recently initiated for behavioural management during his psychiatric admission.

**Figure 1:**
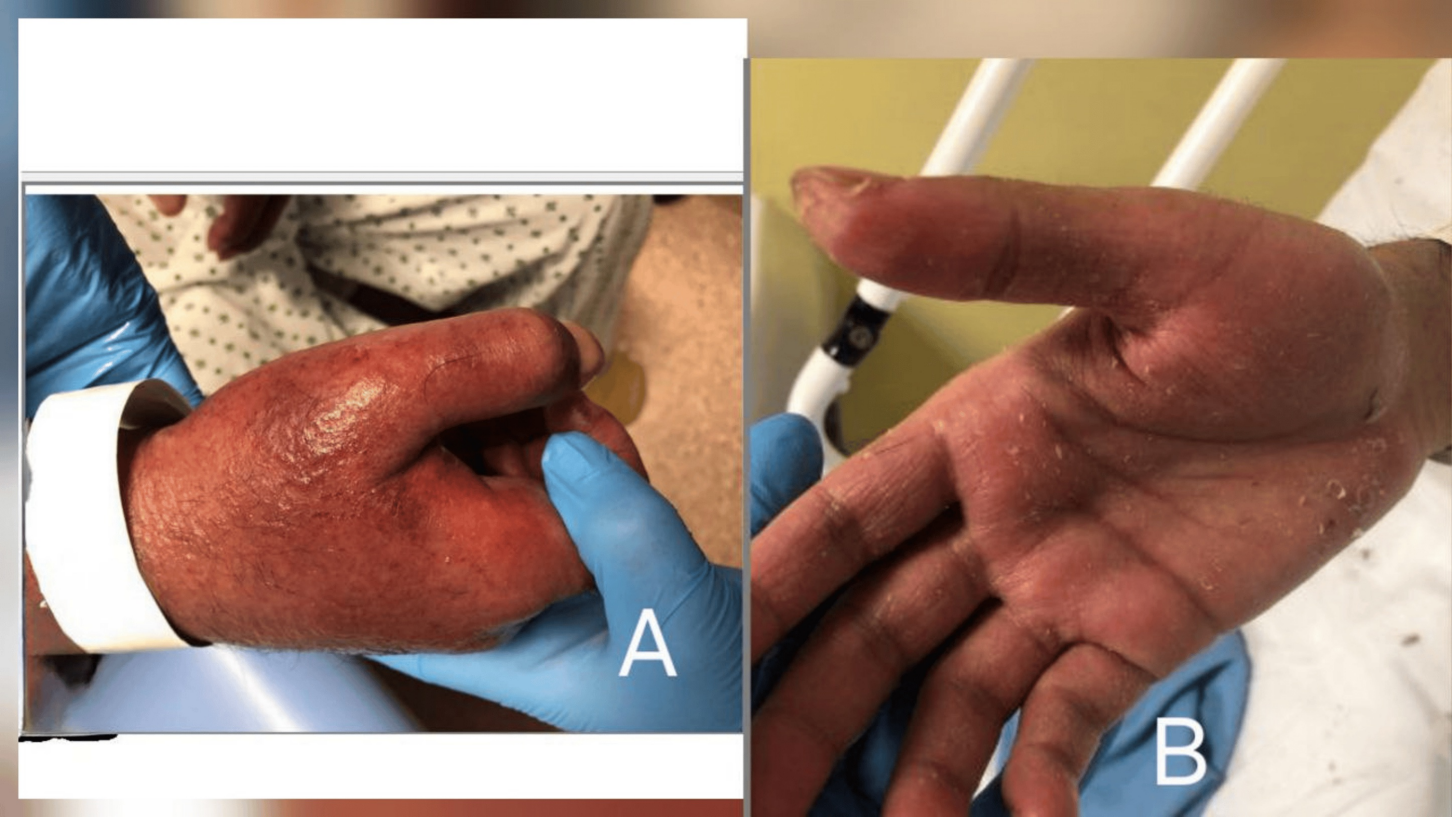
Generalized erythematous pruritic rash on the hand (A). Area of dryness and desquamation on the palmar aspect (B).

**Figure 2:**
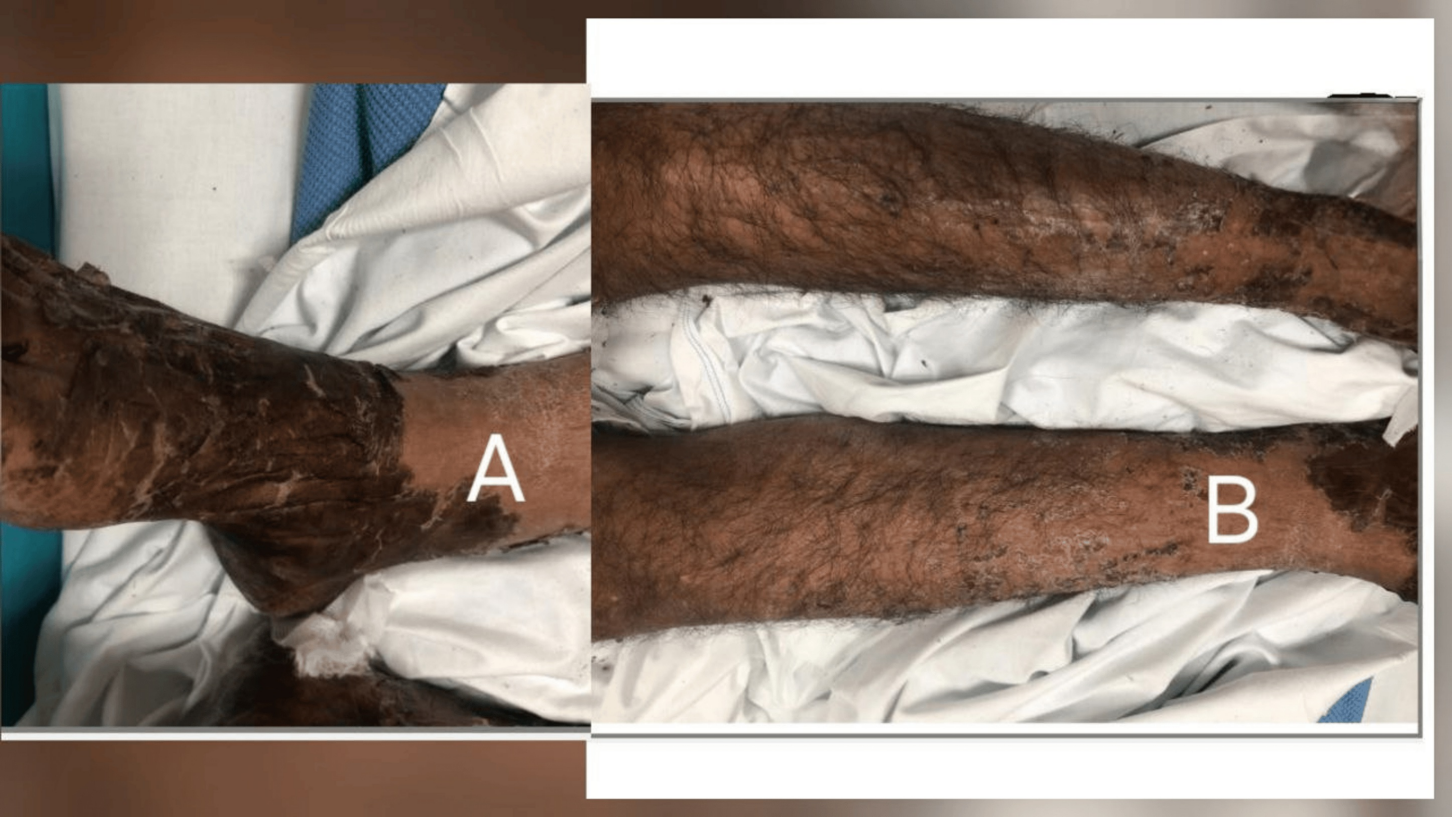
Desquamation of the lower limbs (A). Erythematous scaly patch with a papule on the anterior aspect of the lower limbs (B).

On examination, the patient was febrile (38.1°C), normotensive (BP 134/75 mmHg), and bradycardic (HR 67 bpm). Dermatological examination revealed diffuse erythematous, blistering rash with excoriation affecting the trunk, limbs, and face, with prominent perioral involvement but no mucosal ulceration (Figures 1, 2). Generalized lymphadenopathy was noted, including a palpable right axillary lymph node. Cardiovascular and respiratory examinations were otherwise unremarkable.

Early dermatology review suggested a differential diagnosis including Stevens-Johnson syndrome and severe drug reaction. Features of eczema and seborrhoeic dermatitis were also observed. Serial review, exclusion of alternative diagnoses, and histopathology ultimately indicated DRESS syndrome.

Laboratory blood tests demonstrated leukocytosis (WBC 13.4 × 10^9^/L) on admission, peaking at 63 × 10^9^/L before declining with treatment. Marked eosinophilia was noted (peak 8.24 ×10^9^/L, 22% of total WBC). Atypical lymphocytes were present (peak 10.8 ×10^9^/L). CRP was elevated (peak 113 mg/L) and normalized with therapy. Renal function was preserved (creatinine 77 µmol/L), while liver function tests were initially deranged and improved following treatment (Table 1). Thyroid function was within normal limits. Serology for Epstein-Barr virus was negative.

**Table 1:** Laboratory tests showing extremely high white blood cell count, lymphocytes, and eosinophils.

Test	Result	Normal Range
White Cell Count	63 x 10^9^/L	4-11 x 10^9^/L
Hemoglobin	89 g/L	130-165 g/L
Platelet	477 x 10^9^/L	152-450 x 10^9^/L
Eosinophils	8.29 x 10^9^/​​​​​​​L	0-0.4 x 10^9^/​​​​​​​L
Lymphocytes	10.8 x 10^9^/​​​​​​​L	1-3 x 10^9^/​​​​​​​L
C-reactive Protein	113 mg/L	0-4.9 mg/L
Serum Creatinine	77 µmol/L	40-80 µmol/L

Further investigations, including a core biopsy of a right axillary lymph node, revealed preserved architecture with paracortical expansion, pallor, melanophages, scattered eosinophils, and clusters of Langerhans cells. Immunohistochemistry demonstrated a mixed population of CD4+ and CD8+ T cells, preserved B- and T-cell zones, and reactive germinal centers. These findings were consistent with dermatopathic lymphadenitis and reactive lymphoid hyperplasia.

Based on the patient's presentation of a widespread rash, eosinophilia, systemic involvement, and a recent history of starting carbamazepine, the primary differential diagnosis included DRESS syndrome, which was favored over viral exanthema, Stevens-Johnson syndrome (due to the lack of significant mucosal involvement), and lymphoproliferative disorders (ruled out by lymph node biopsy). Utilizing the European Registry of Severe Cutaneous Adverse Reactions (RegiSCAR) scoring system, the diagnosis was confirmed as DRESS syndrome secondary to carbamazepine.

Carbamazepine was immediately discontinued. The patient was commenced on intravenous hydrocortisone, followed by a tapering course of systemic corticosteroids. Supportive management included antipyretics, antihistamines for pruritus, emollients, and close monitoring of liver and renal function. Dermatology and psychiatry teams were involved throughout.

Following drug withdrawal and corticosteroid therapy, the patient’s fever resolved, and the cutaneous eruption improved. Lymphadenopathy remained stable. The patient continues follow-up with dermatology and psychiatry.

## Discussion

DRESS syndrome is a potentially fatal hypersensitivity reaction with a heterogeneous clinical presentation. Carbamazepine is among the most frequently implicated agents [6]. Delayed onset after drug exposure can complicate diagnosis. Hallmark features include fever, rash, eosinophilia, lymphadenopathy, and internal organ involvement, particularly of the liver and kidneys [7].

Diagnosis is primarily clinical, supported by laboratory and histopathological findings. Dermatopathic lymphadenitis, as in this case, is a benign reactive process commonly associated with extensive skin disease and supports the diagnosis of DRESS [4,8]. Prompt discontinuation of the offending agent is essential, and systemic corticosteroids are recommended for significant systemic involvement [1,9]. Multidisciplinary care is critical in patients with complex psychiatric comorbidities to ensure safe management and medication rationalization.

## Conclusions

High clinical suspicion for DRESS syndrome should be maintained in patients presenting with rash and systemic symptoms after starting high-risk medications such as carbamazepine. Early recognition, prompt drug cessation, and coordinated multidisciplinary care are crucial to reducing morbidity and improving outcomes.

## References

[1] Cacoub P, Musette P, Descamps V, Meyer O, Speirs C, Finzi L, Roujeau JC (2011). The DRESS syndrome: a literature review. Am J Med.

[2] Bocquet H, Bagot M, Roujeau JC (1996). Drug-induced pseudolymphoma and drug hypersensitivity syndrome (drug rash with eosinophilia and systemic symptoms: DRESS). Semin Cutan Med Surg.

[3] Kardaun SH, Sidoroff A, Valeyrie-Allanore L, Halevy S, Davidovici BB, Mockenhaupt M, Roujeau JC (2007). Variability in the clinical pattern of cutaneous side-effects of drugs with systemic symptoms: does a DRESS syndrome really exist?. Br J Dermatol.

[4] Chen YC, Chiu HC, Chu CY (2010). Drug reaction with eosinophilia and systemic symptoms: a retrospective study of 60 cases. Arch Dermatol.

[5] Husain Z, Reddy BY, Schwartz RA (2013). DRESS syndrome: part I. Clinical perspectives. J Am Acad Dermatol.

[6] E L omairi N, Abourazzak S, Chaouki S, Atmani S, Hida M (2014). Drug reaction with eosinophilia and systemic symptom (DRESS) induced by carbamazepine: a case report and literature review. Pan Afr Med J.

[7] Calle AM, Aguirre N, Ardila JC, Cardona Villa R (2023). DRESS syndrome: a literature review and treatment algorithm. World Allergy Organ J.

[8] Mantri N, Qasim A, Zacharia GS, Hoazhe S, Patel H (2025). Carbamazepine-induced drug rash with eosinophilia and systemic symptoms (DRESS) syndrome. Cureus.

[9] Ilahi HB, Baloch MB, Kasfiki E (2025). Drug reaction with eosinophilia and systemic symptoms (DRESS) due to trimethoprim: a case report. Cureus.

